# Porcelain Laminate Veneers: A Case Report

**DOI:** 10.7759/cureus.34220

**Published:** 2023-01-26

**Authors:** Dhanashree A Minase, Seema Sathe, Anjali Bhoyar, Chinmayee Dahihandekar, Tanvi Jaiswal

**Affiliations:** 1 Prosthodontics Crown and Bridge, Sharad Pawar Dental College and Hospital, Datta Meghe Institute of Medical Sciences, Wardha, IND

**Keywords:** conservative, anterior restoration, laminates, smile designing, esthetics in dentistry

## Abstract

One of the most crucial issues in modern dentistry is restoring a patient's lost dental aesthetic appearance. To do this, new therapeutic tools and techniques have been appearing on the market day by day. For anterior teeth, where aesthetics are extremely essential, the majority of dentists prefer more conservative and aesthetic treatments, such as direct and indirect laminate veneer restorations over full-ceramic crowns. The meteoric emergence of veneers has been one of dentistry's most successful innovations. It is a coating of tooth-coloured material that is put to a tooth to aesthetically restore any intrinsic discoloration or localised or generalised abnormalities. Any restoration work ought to be created using mechanical, biological, and aesthetic criteria. Patients may experience significant aesthetic issues as a result of the colour, form and structural and positional anomalies of their anterior teeth. This is a report depicting a case of porcelain laminate veneers using computer-aided design/computer-aided manufacturing (CAD/CAM).

## Introduction

Porcelain laminate veneers (PLVs) have replaced crowns made of ceramics and conventional porcelain which is fused-to-metal as the more aesthetically pleasing option [1,2]. Laminates can successfully modify smiles in a rapid, painless, and conservative manner with effects that last. The completed surface of laminates closely resembles the surface of natural teeth, and they exhibit good tissue response. Veneers have a natural fluorescence and precisely mimic the structure of actual teeth in terms of how they absorb, reflect, and transmit light [1-3]. For rehabilitation of compromised dentition in anterior region the preferred restoration is PLVs such as congenital as well as acquired malformations where dentino-enamel junction is not alveolar, coronal fractures, when anterior teeth require major morphologic modifications such as conoid teeth, diastemas, to increase length of the incisal edge of the tooth and where discolored teeth don't react to bleaching such as degree III and degree IV tetracycline stains [3-5]. PLVs can be considered a new, conservative, esthetic treatment option that requires less tooth preparation and less amount of treatment span. The uniqueness of this case report is that this prosthesis was made from computer-aided design/computer-aided manufacturing (CAD/CAM) technique with cut back technique at the incisal edge.

## Case presentation

A 25-year-old male patient reported to the prosthodontics department at Sharad Pawar Dental College in Sawangi, Wardha, with complaints of aesthetic issues associated with spacing between his top front teeth for 20 years.

Treatment planning

**Figure 1:**
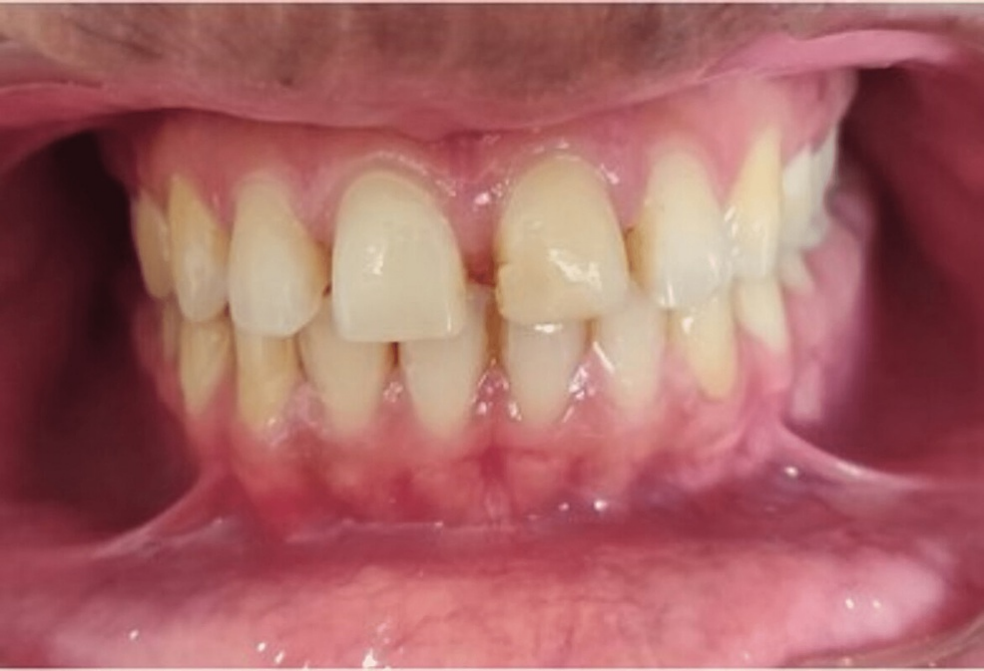
Pre-operative intraoral photograph

**Figure 2:**
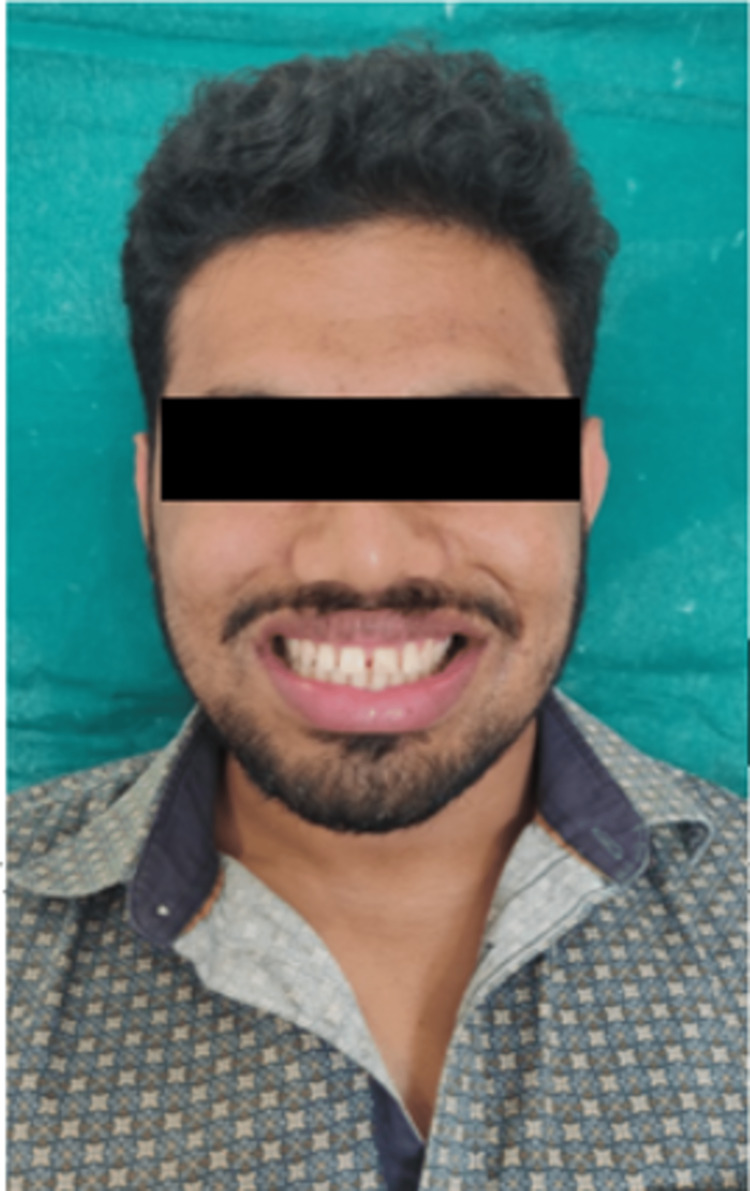
Pre-operative extraoral photograph

**Figure 3:**
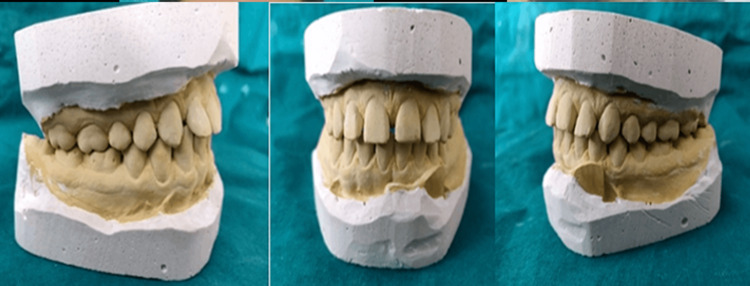
Diagnostic study model

A thorough case history of the patient was recorded. Pre-operative intraoral (Figure 1) and extraoral photographs (Figure 2) were taken. Diagnostic impressions were made and diagnostic study models were prepared (Figure 3).

**Figure 4:**
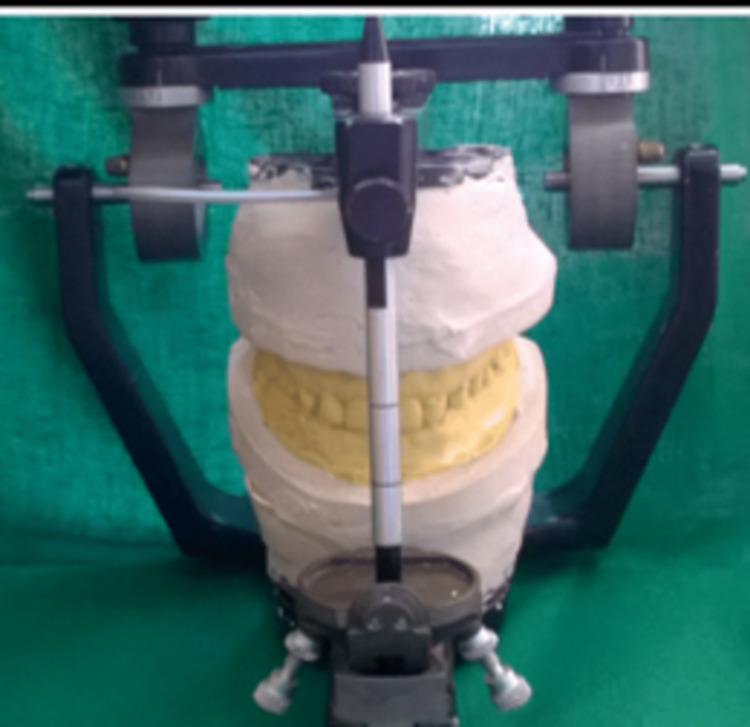
Mounting of maxillary and mandibular rims

**Figure 5:**
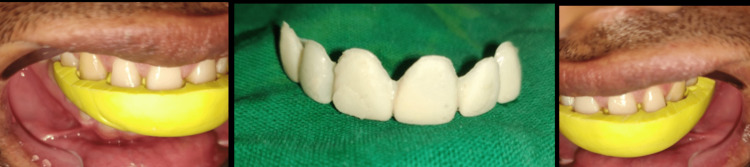
Aesthetic pretempering

After the evaluation of the study models, radiographic evaluation was carried out. Various treatment plans were designed and discussed with the patient. The patient was given the option of receiving both orthodontic and restorative care. He refused the idea of using orthodontic devices, therefore laminate veneers were decided upon instead. A proper consent was taken from the patient before the start of the treatment. On the second visit, facebow record was made and diagnostic mounting was done (Figure 4). After mounting, mock preparation and wax up was carried out (Figure 5).

The primary diagnostic tool to assess differences between present and perfect tooth proportions is wax-up. Provisionalisation was done with a self-cured temporary composite material in order to assess the future dimensions of the proposed ceramic restorations. This step gave us the idea about the amount of tooth preparation that is necessary for this patient.

Tooth preparation

**Figure 6:**
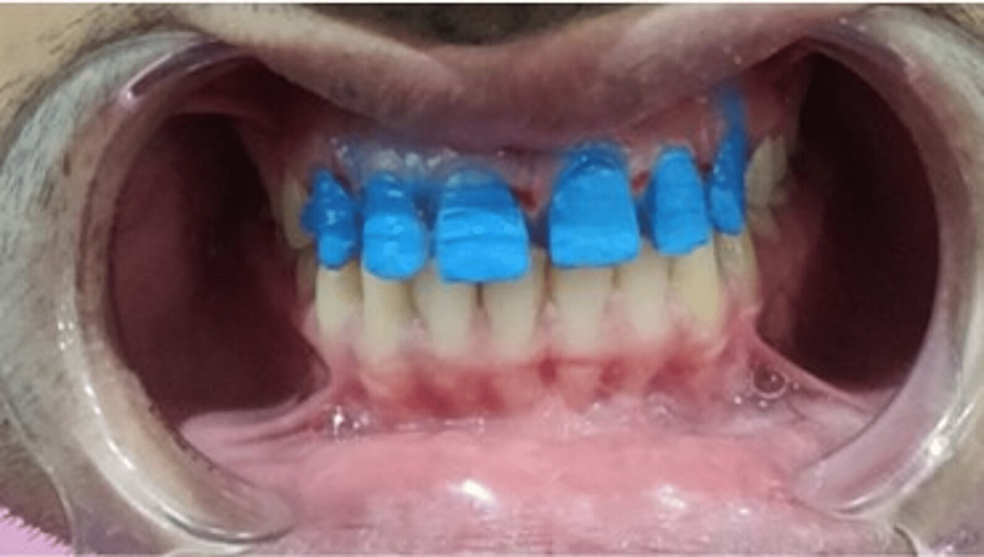
Tooth preparation

**Figure 7:**
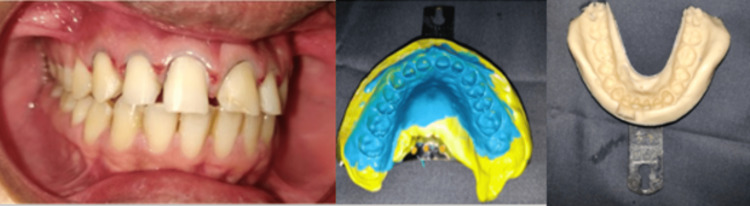
Impressions

On the third visit tooth preparations were carried out. By setting a cervical step without obstructing the natural gingival contour, the preparations' cervical borders were positioned right at the gingiva's level (equigingival). The chamfer finish line was preferred for the preparation. Care was taken to round off all the internal line angles to reduce stresses in the margins of the veneers. Incisal overlap preparation was performed (Figure 6) and the teeth were polished and smoothed. Further final impression with polyvinyl siloxane as a wash impression was made after gingival retraction using double step impression technique (Figure 7).

**Figure 8:**
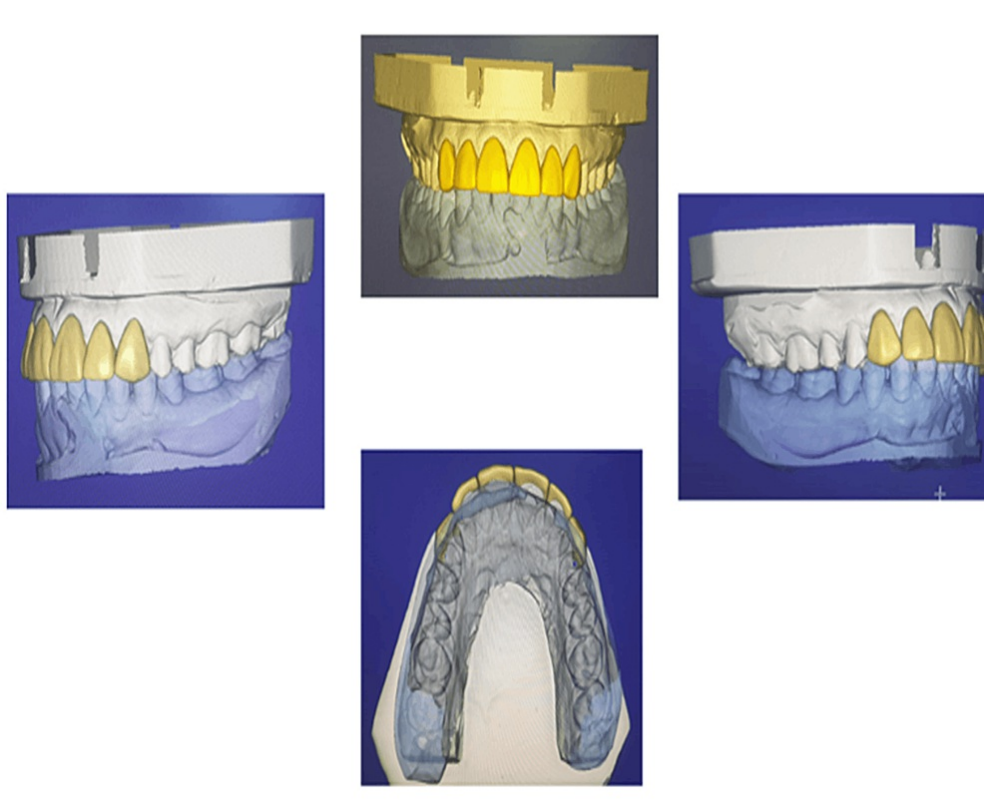
CAD/CAM designing

The shade was selected with VITA 3D master shade guide under natural light. Using shade tabs, we carried out shade selection, a crucial step in our treatment protocol, at the beginning of the visit before we prepared the teeth. The other methods include reference photography and technology-based systems. Temporary restoration was given using Luxa Temp Ultra DMG. After fabrication of the master cast scanning of cast was done using Dentsply Sirona Scanner followed by CAD/CAM designing and milling of Emax Prosthesis (Figure 8).

Cementation procedure

**Figure 9:**
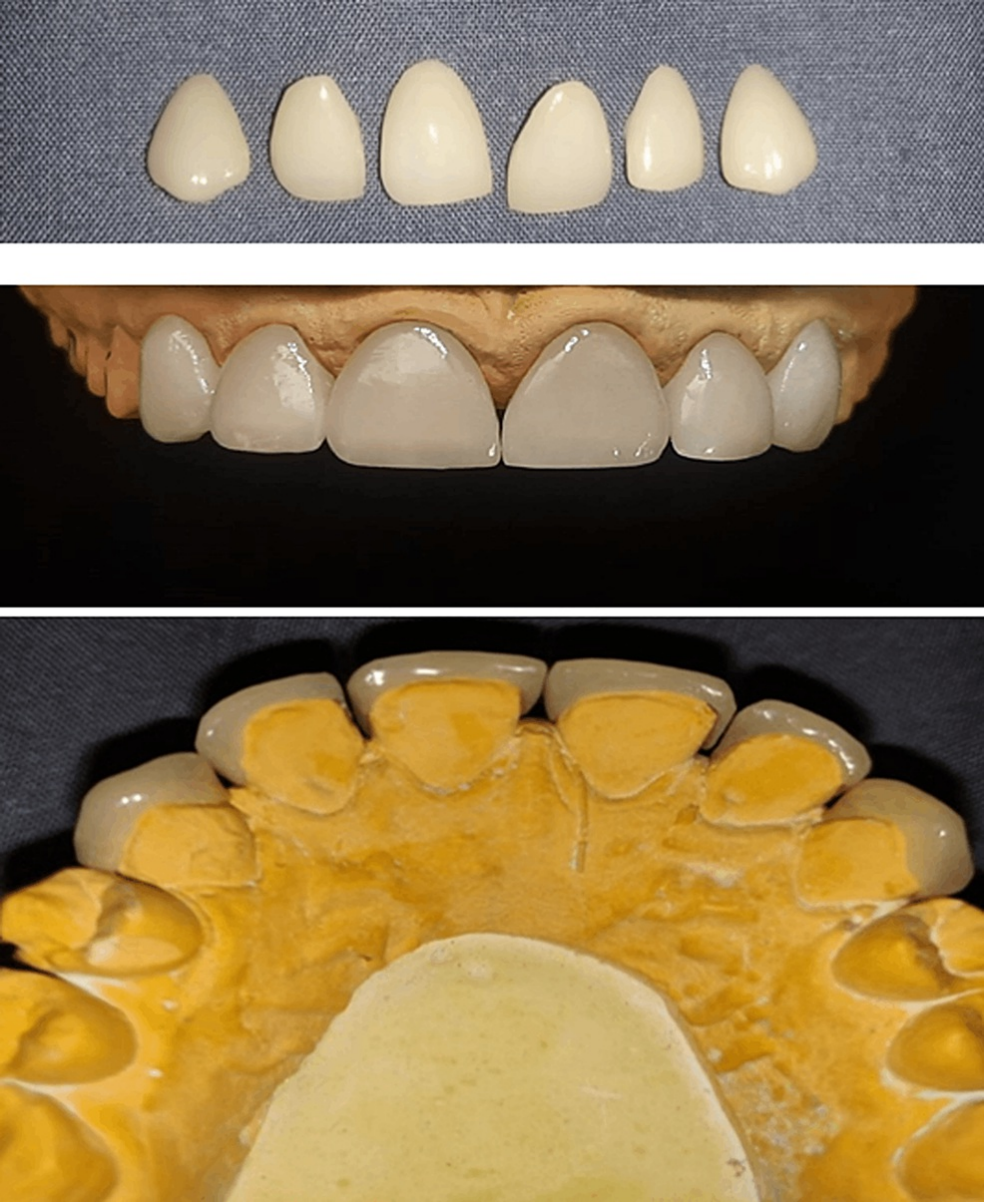
Final prosthesis

On the fourth visit, temporary veneers were removed followed by cleaning and isolation of the teeth. The quality of fit, gingival extension and color match of the veneer was assessed. All six laminates were tried by placing them one by one to check adaptation and alignment. Adhesive techniques must be done in a completely isolated environment under rubber dam before bonding the final restorations. Cementation of EMAX veneers was performed by following the proper cementation protocol (Figure 9).

Treatment surface of the laminate veneers and tooth

**Figure 10:**
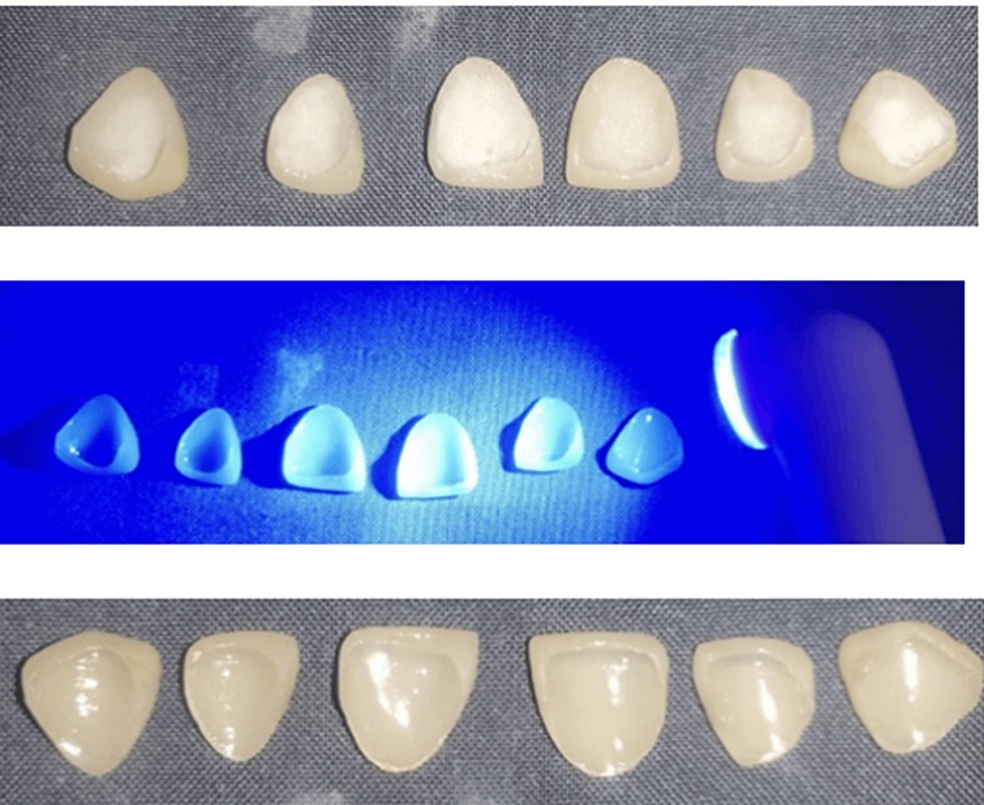
Visible light curing

**Figure 11:**
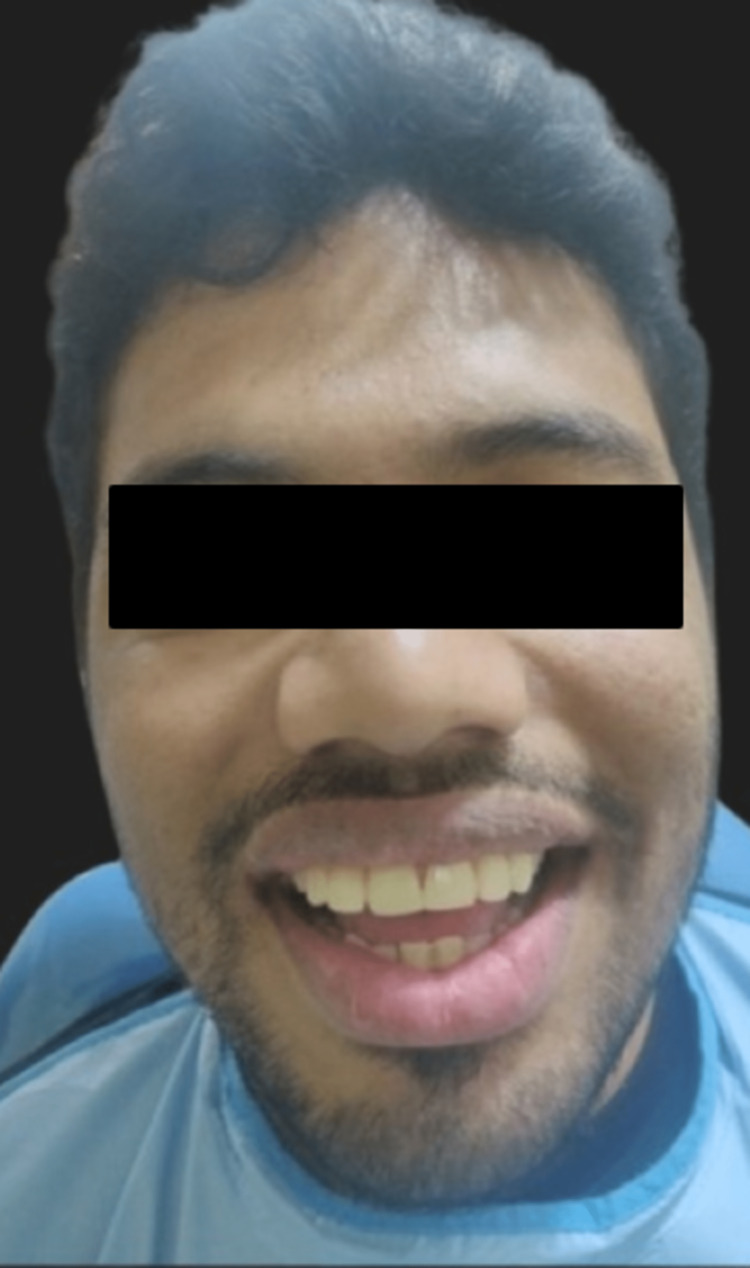
Postoperative photograph

The intaglio surface of the veneers was etched using 30% hydrofluoric gel, rinsed and coated with a silane coupling agent. The prepared tooth was well isolated and etched with 37% orthophosphoric acid (Universal Etch), rinsed and Prime & Bond NT dentin bonding agent was applied following the manufacturer’s instructions. Calibra (Dentsply) resin luting cement was used for the cementation of the porcelain laminate veneers. The luting resin was then cured using a visible light activation device for 40 seconds each after all gross surplus had been eliminated (Figure 10). PLVs were finished using rotating abrasive disks (Soflex discs). The patient was given oral hygiene and home care instructions for adequate care. A postoperative photograph of the patient is shown in Figure 11.

## Discussion

Selection of the patient is important for success of PLVs. In the present case because of the young age of the patient and the unwillingness to undergo extensive orthodontic treatment by the patient, a conservative method of treatment by PLVs was selected. PLVs were deemed to be the most preferable treatment option since they had a favorable smile line, were parafunctional-free, and had enough enamel. The benefits of using these restorations include their biological acceptability to the body due to their increased chemical stability, less cytotoxicity, decreased risk of causing irritation or sensitivity, stability, and high resistance against abrasion. They also have a conservative approach and prevent excessive removal of natural teeth [6]. Due to their glazed surface which is uniform, these restorations display decreased build-up of plaque and its simple removal. The PLVs are fracture-prone even before they are cemented because of the ceramic's thinness (0.3-0.5mm). But once they are bound to the etched enamel surface, they blend in with the tooth structure and grow much more resilient. A long-lasting restoration is produced by joining porcelain and etched enamel, as well as by bonding composite resin-luting agent and silane coupling agent. When enamel is weak, parafunction habits like bruxism or clenching, inappropriate anatomical presentation of teeth PLVs should be avoided. Bonding onto the composite restorations which is pre-existing, an untrained practitioner and use of veneers to repair worn-down teeth with significant dentin exposure and remaining tooth structure are risk factors for veneer failure. The tendency for heat fluctuations to result in cracking of the veneer when the luting composite is thick and the porcelain is thin as demonstrated by in-vitro research, is another risk factor. A thick composite layer may develop as a result of a veneer that does not fit properly or as a result of using a lot of die spacer to cover up underlying tooth discoloration. A ceramic and luting composite thickness ratio greater than 3 resulted in the least amount of cracking.

To conceal tooth discoloration or unattractive shapes, composite restorations may be used instead of porcelain laminate veneers. Enamel hypoplasia, midline diastema and the occurrence of microdontically unusually shaped teeth such as peg laterals are common aesthetic issues in young people that are still conservatively addressed with porcelain laminate veneers [7]. Composites are prone to wear, marginal fractures and discoloration, hence their durability is debatable. For simple and sophisticated teeth-supported restorations, CAD/CAM solutions provide a standardized manufacturing process that results in a dependable, predictable and cost-effective workflow. Therefore, clinicians must consider CAD/CAM technology and it will be very useful in the near future as it is time-saving. To confirm the clinical performance of minimally invasive and extensive restorations supported by zirconia teeth and monolithic polymer-infiltrated ceramic networks, more data from long-term clinical trials is required.

## Conclusions

As there was sufficient enamel height, no parafunctional habit history, vital tooth and good oral hygiene, PLVs were the best treatment option for the case instead of orthodontic treatment or composite restoration which can get discoloured over a period of time. Combining CAD/CAM and PLVs can be considered for better aesthetic results as it is a new, conservative, aesthetic treatment option that requires less tooth preparation and less amount of treatment span. Along with these advantages, nature appearance of the tooth was restored. Use of CAD/CAM software decreases chair side time and enhances prosthesis design in less time. So, use of this software should be encouraged by clinicians.
